# Weanling Offspring of Dams Maintained on Serine-Deficient Diet Are Vulnerable to Oxidative Stress

**DOI:** 10.1155/2018/8026496

**Published:** 2018-09-16

**Authors:** Liuqin He, Haiwen Zhang, Xihong Zhou

**Affiliations:** ^1^Hunan International Joint Laboratory of Animal Intestinal Ecology and Health, Laboratory of Animal Nutrition and Human Health, College of Life Sciences, Hunan Normal University, Changsha 410081, China; ^2^Key Laboratory of Tropical Animal Breeding and Epidemic Disease Research of Hainan Province, Hainan University, Haikou 570228, China; ^3^Key Laboratory of Agro-Ecological Processes in Subtropical Region, Institute of Subtropical Agriculture, Chinese Academy of Sciences, Changsha 410125, China

## Abstract

Serine plays an important role in the antioxidant defense system. However, the effects of maternal serine deficiency on the antioxidant ability of weanling offspring have not been reported. In the present study, we investigated the oxidative status of offspring of dams that are maintained on serine-deficient diet and subjected to diquat challenge. Individual pregnant animals were randomly divided into two dietary groups, namely, the control diet group and the serine- and glycine-deficient diet group. Samples were collected from weanling offspring at the age of 3 weeks after diquat challenge. Our results showed that maternal serine deficiency did not affect the levels of antioxidant enzymes and reactive oxygen species, as well as the expression of cellular and mitochondrial stress markers (*Hspd1* and *Hspa1a*), which indicated that maternal serine deficiency did not affect basal oxidative status in weanling offspring. However, the weanling offspring were found to be vulnerable to oxidative challenges. Furthermore, our results suggested that the dysfunctional antioxidant system in response to oxidative stress in offspring of dams fed with serine-deficient diet was primarily caused by reduced availability of nicotinamide adenine dinucleotide phosphate. Furthermore, impairment of the antioxidant defense system caused by maternal serine deficiency was mediated by the Akt/AMPK/Sirt1 pathway. Our results indicated that maternal serine availability is important for maintaining antioxidant defense against oxidative challenge in weanling offspring.

## 1. Introduction

Maternal nutrition is strongly associated with offspring health. A suboptimal in utero environment often leads to higher rates of the development of cardiovascular and metabolic diseases in the offspring [1], which are in turn closely associated with oxidative stress. Continuous adequate or overnutrition under postnatal conditions could not reverse the detrimental effects caused by maternal malnutrition [2].

Serine is part of a metabolic network that links the one-carbon cycle to glycolysis to support oxidant/antioxidant balance and cell proliferation [3, 4]. Serine exerts its antioxidant effects through various molecular pathways. Serine is a precursor of glycine and cysteine, both of which are major substrates required for the synthesis of cellular antioxidant glutathione (GSH). Moreover, the increased levels of GSH promote the S-glutathionylation of the AMP-activated protein kinase *α* subunit (AMPK*α*) [5] and activate the AMPK signaling pathway, which is crucial for mediating the adaptive response to oxidative stress. In addition, Akt and Sirt1 which are involved in the AMPK signaling pathway play important roles in oxidative stress [6]. Akt is a redox-regulated protein which is directly regulated by the GSH redox system [7]. Sirt1 is also a redox-sensitive deacetylase, and Sirt1 protein expression is generally related to the GSH level [8]. However, whether Akt and Sirt1 are affected by serine deficiency remains unknown.

Recent studies showed that serine is an important component for the synthesis of NADPH, a major cellular reducing agent [9–11]. The de novo serine synthesis pathway plays critical roles in metabolic processes, such as cell growth and cell survival. In particular, the de novo serine synthesis is crucial for maintaining redox homeostasis in both the cellular and mitochondrial levels [12, 13]. The recent study demonstrated that the inhibition of de novo serine synthesis leads to mitochondrial dysfunction and impaired oxidative stress response [14]. However, our previous findings suggested that exogenous serine ameliorates oxidative damage in various tissues in mice and in different models subjected to various kinds of oxidative stressors [5, 15–17]. In addition, the previous study showed that serine starvation increased the sensitivity of cells to peroxide treatment [18], which suggested that exogenous serine is important for maintaining cellular antioxidant capacity. However, to the best of our knowledge, the effects of maternal serine deficiency on the antioxidant ability of offspring have not been reported. Diquat has long been used to induce oxidative damage primarily in the liver in animal models [15]. It can be converted to cation free radical which reacts rapidly with molecular oxygen. This reaction produces lots of superoxide anion radical and causes a rise in the glutathione redox ratio. Consequently, we conducted the present study to investigate the oxidative status of offspring of dams maintained on serine-deficient diet and subjected to diquat challenge.

## 2. Materials and Methods

### 2.1. Animal Care and Experimental Design

C57BL/6J mice (10 weeks old) were purchased from the SLAC Laboratory Animal Central (Changsha, China). After 1 week of acclimatization, all animals were fed with control diet and mated and the females were then checked for the presence of postcopulatory plugs. Pregnant animals were randomly divided into two dietary groups, namely, the control (CON) diet group (Research Diets, New Brunswick, NJ, USA) and the serine- and glycine-deficient (SGD) diet group (no added serine and glycine; Research Diets). The components of the diet are presented in Supplementary Table 1. Dams from each group were randomly assigned to the treatment groups with eight offspring per group (four males and four females). All mice were housed under standard conditions in pathogen-free colonies (temperature, 22 ± 2°C; relative humidity, 50 ± 5%; lighting cycle, 12 h/d) and were provided with free access to food and water. At the age of 3 weeks, male and female mice were randomly chosen from each dam and injected intraperitoneally with either diquat at 12 mg/kg body weight or equal saline. At 3 hours after the injection, blood was collected from the retroorbital sinus. Afterwards, mice were sacrificed by cervical dislocation, and liver samples were collected for further analysis. All the procedures in the present study were approved by the Animal Welfare Committee of the Institute of Subtropical Agriculture, Chinese Academy of Sciences. All procedures were carried out according to the rules established by the committee.

### 2.2. Determination of Malondialdehyde, Glutathione, Superoxide Dismutase, and Catalase Contents and Glutathione S-Transferase Activity

Malondialdehyde (MDA), glutathione (GSH), superoxide dismutase (SOD), and catalase (CAT) contents in serum and liver were analyzed using commercial kits according to the manufacturer's instructions (Northwest Life Science Specialties, Vancouver, WA, USA). Glutathione S-transferase (GST) activity was measured using a commercial kit according to the manufacturer's instructions (Cayman Chemical Company, Ann Arbor, Michigan, USA).

### 2.3. Determination of Serine and Glycine Content

After pretreatment according to the previous study [19], the liver sample (100 mg) or serum sample (0.2 mL) was mixed with 1 mL 10% sulfosalicylic acid and then maintained at 4°C for 5 min. After centrifugation at 13000 rpm for 15 min, the supernatant was filtered through 0.22 *μ*m filters and then analyzed by an ion-exchange amino acid analyzer (L8800, Hitachi, Tokyo, Japan).

### 2.4. Determination of Complex I and III Activities

Mitochondria were isolated from liver samples using a commercial kit (Solarbio, Beijing, China). Afterwards, complex I (NADH ubiquinone oxidoreductase) and complex III (ubiquinol cytochrome reductase) activities were assessed spectrophotometrically using commercial kits according to the manufacturer's instructions (Solarbio).

### 2.5. Determination of Reactive Oxygen Species Levels

Reactive oxygen species (ROS) content was analyzed as previously described [15]. Briefly, fresh samples were immediately embedded in a tissue-freezing medium (Sakura, Tokyo, Japan) and frozen in a methylbutane-chilled bath at −80 ± 2°C. Sections (10 *μ*m) were stained with 1 *μ*M dihydroethidium (Sigma-Aldrich, Shanghai, China) for 20 min at 37°C. Images were obtained and analyzed using Image Browser software (Leica, Wetzlar, Germany).

### 2.6. Determination of Lipid Hydroperoxide and NADPH Contents

Lipid hydroperoxide (LPO) content in the liver sample was determined spectrophotometrically (Northwest Life Science Specialties). Nicotinamide adenine dinucleotide phosphate (NADPH) level was measured using a NADP^+^/NADPH Quantification kit (BioVision, Milpitas, CA, USA).

### 2.7. RT-qPCR Analysis

Liver sample was ground, and total RNA was isolated using the TRIzol reagent (Invitrogen, Carlsbad, CA, USA). The PrimeScript RT reagent kit (Takara, Dalian, China) was used for reverse transcription of total RNA. RT-qPCR was performed in 10 *μ*L assay volumes containing 5 *μ*L of SYBR Green mix (Takara), 0.2 *μ*L of ROX, 3 *μ*L of diethylpyrocarbonate-treated deionized H_2_O, 1 *μ*L of cDNA template, and 0.4 *μ*L each of the forward and reverse primers [20]. The primer sequences used for RT-qPCR are presented in Supplementary Table 2. All samples were run in triplicate, and the average values were calculated.

### 2.8. Western Blotting Analysis

Western blotting analysis was performed for the determination of relative protein levels of Akt, Sirt1, AMPK, and nrf2. Briefly, 20 *μ*g of protein per lane was separated by SDS-PAGE and blotted onto nitrocellulose membranes. Membranes were incubated with primary antibodies against AMPK*α*, phosphorylated AMPK*α*, nuclear factor-like 2 (nrf2), Sirt1, Akt, phosphorylated Akt (Cell Signaling, Beverly, MA, USA), and *β*-actin (Boster, Wuhan, China) overnight at 4°C. Then, membranes were incubated overnight with the secondary antibodies and subsequently exposed to EZ-ECL (Biological Industries, Cromwell, CT, USA).

### 2.9. Statistical Analysis

Statistical analysis was performed by factorial ANOVA using a mixed procedure (PROCMIXED) in SAS software version 9.2 (SAS Institute Inc., Cary, NC, USA). The effects of diet (control or SGD diet), challenge (diquat or saline), and their interactions were incorporated into the statistical model. Data were presented as least square means plus pooled SEM. *P* < 0.05 was considered statistically significant.

## 3. Results

### 3.1. Effects of Maternal Serine Deficiency on SOD, GSH, CAT, MDA, Serine, and Glycine Levels and GST Activity in Weanling Offspring Subjected to Diquat Challenge

**Figure 1:**
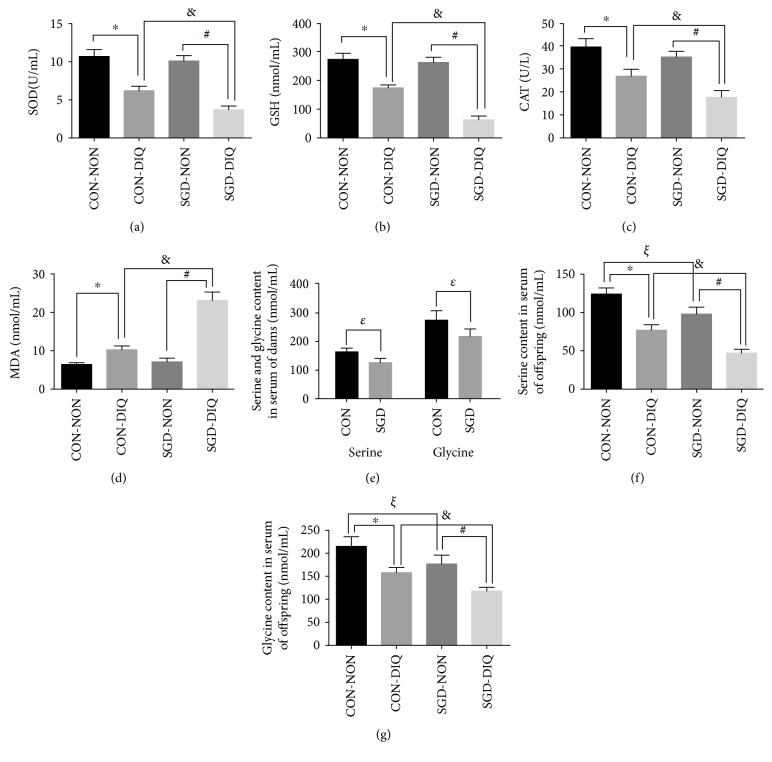
SOD, GSH, CAT, MDA, serine, and glycine contents in serum. SOD: superoxide dismutase; GSH: glutathione; CAT: catalase; MDA: malondialdehyde; CON-NON: offspring from dams fed the basal diet; CON-DIQ: offspring from dams fed the basal diet and subjected to diquat challenge; SGD-CON: offspring from dams fed with serine-deficient diet; SGD-DIQ: offspring from dams fed with serine-deficient diet and subjected to diquat challenge. Values are expressed as LSmean plus pooled SEM, *n* = 8. ^∗^Mean values were significantly different between CON-NON and CON-DIQ (*P* < 0.05). ^#^Mean values were significantly different between SGD-NON and SGD-DIQ (*P* < 0.05). ^&^Mean values were significantly different between CON-DIQ and SGD-DIQ (*P* < 0.05). ^ξ^Mean values were significantly different between CON-NON and SGD-NON (*P* < 0.05). ^ε^Mean values were significantly different between CON and SGD in dams (*P* < 0.05).

**Figure 2:**
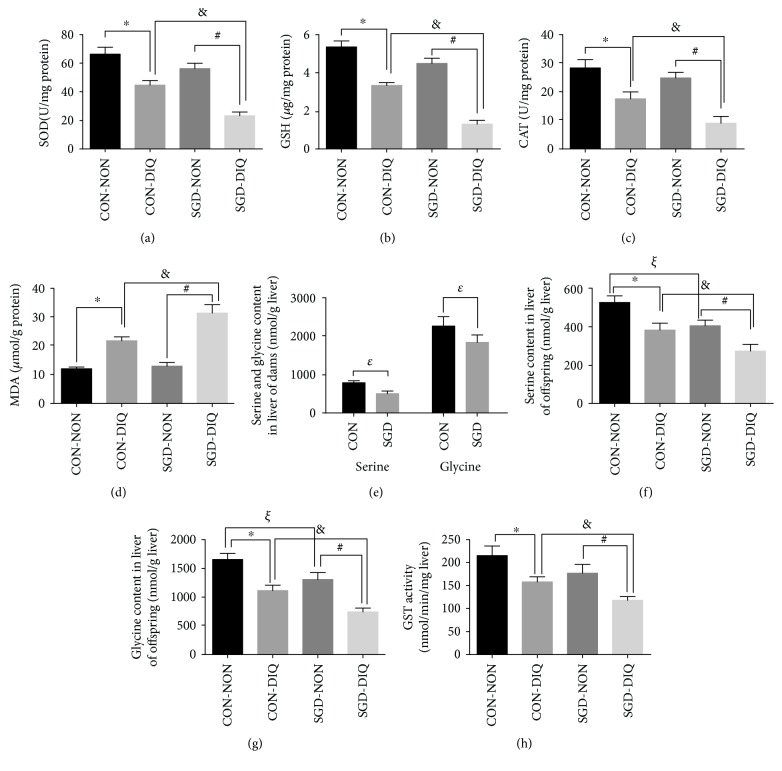
SOD, GSH, CAT, MDA, serine and glycine contents, and GST activity in the liver. SOD: superoxide dismutase; GSH: glutathione; CAT: catalase; MDA: malondialdehyde; GST: glutathione S-transferase; CON-NON: offspring from dams fed the basal diet; CON-DIQ: offspring from dams fed the basal diet and subjected to diquat challenge; SGD-CON: offspring from dams fed with serine-deficient diet; SGD-DIQ: offspring from dams fed with serine-deficient diet and subjected to diquat challenge. Values are expressed as LSmean plus pooled SEM, *n* = 8. ^∗^Mean values were significantly different between CON-NON and CON-DIQ (*P* < 0.05). ^#^Mean values were significantly different between SGD-NON and SGD-DIQ (*P* < 0.05). ^&^Mean values were significantly different between CON-DIQ and SGD-DIQ (*P* < 0.05). ^ξ^Mean values were significantly different between CON-NON and SGD-NON (*P* < 0.05). ^ε^Mean values were significantly different between CON and SGD in dams (*P* < 0.05).

Our results showed that maternal serine deficiency did not affect SOD, GSH, CAT, and MDA levels in both serum (Figure 1) and liver (Figure 2) samples of weanling offspring. However, under diquat challenge, offspring from dams fed with serine-deficient diet showed significantly lower SOD, GSH, and CAT levels in both serum and liver samples and significantly higher MDA levels relative to those of the offspring of dams fed with the basal diet. Serine and glycine levels as well as GST activity were decreased under diquat challenge. Dams fed with serine-deficient diet and their offspring showed significantly lower serine and glycine levels in both serum and liver samples when compared with dams fed with the basal diet or their offspring, respectively.

### 3.2. Effects of Maternal Serine Deficiency on the Expression of Gpx, SOD, and CAT in Weanling Offspring Subjected to Diquat Challenge

**Figure 3:**
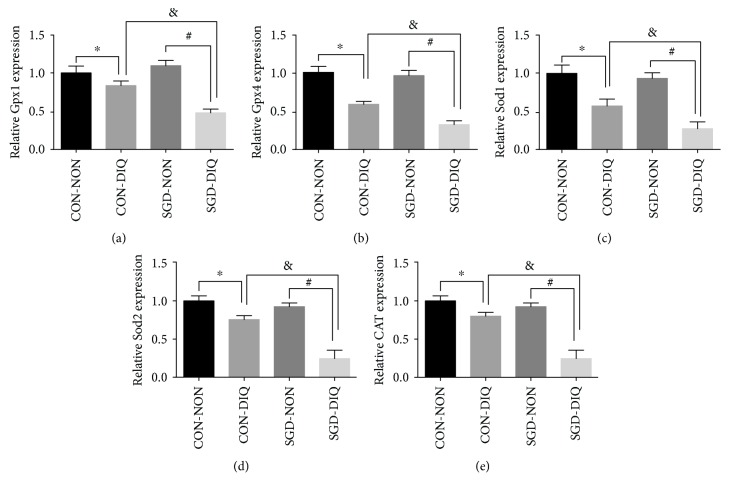
Gene expression of *Gpx*, *Sod*, and *Cat* in the liver. Gpx: glutathione peroxidase; SOD: superoxide dismutase; CAT: catalase; CON-NON: offspring from dams fed the basal diet; CON-DIQ: offspring from dams fed the basal diet and subjected to diquat challenge; SGD-CON: offspring from dams fed with serine-deficient diet; SGD-DIQ: offspring from dams fed with serine-deficient diet and subjected to diquat challenge. Values are expressed as LSmean plus pooled SEM, *n* = 8. ^∗^Mean values were significantly different between CON-NON and CON-DIQ (*P* < 0.05). ^#^Mean values were significantly different between SGD-NON and SGD-DIQ (*P* < 0.05). ^&^Mean values were significantly different between CON-DIQ and SGD-DIQ (*P* < 0.05).

As shown in Figure 3, maternal serine deficiency did not affect the expression of Gpx1, Gpx2, Sod1, Sod2, and CAT in weanling offspring. However, under diquat challenge, offspring of dams fed with the serine-deficient diet showed a significant downregulation of Gpx1, Gpx2, Sod1, Sod2, and CAT levels relative to those of the offspring of dams fed with the basal diet.

### 3.3. Effects of Maternal Serine Deficiency on Mitochondrial Complex I and III Activities in Weanling Offspring Subjected to Diquat Challenge

**Figure 4:**
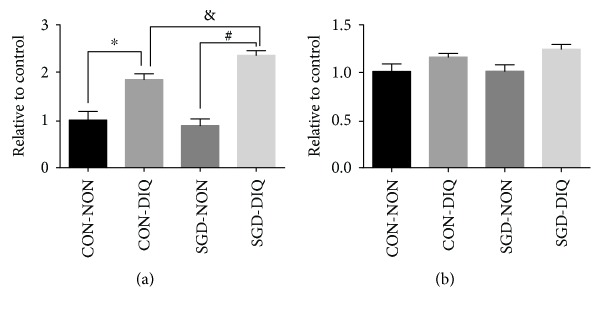
Activities of mitochondrial complexes I and III. (a) Complex I activity. (b) Complex III activity. CON-NON: offspring from dams fed the basal diet; CON-DIQ: offspring from dams fed the basal diet and subjected to diquat challenge; SGD-CON: offspring from dams fed with serine-deficient diet; SGD-DIQ: offspring from dams fed with serine-deficient diet and subjected to diquat challenge. Values are expressed as LSmean plus pooled SEM, *n* = 8. ^∗^Mean values were significantly different between CON-NON and CON-DIQ (*P* < 0.05). ^#^Mean values were significantly different between SGD-NON and SGD-DIQ (*P* < 0.05). ^&^Mean values were significantly different between CON-DIQ and SGD-DIQ (*P* < 0.05).

As shown in Figure 4, maternal serine deficiency did not influence complex I and III activities in weanling offspring. However, under diquat challenge, offspring of the dams fed with the serine-deficient diet showed significantly higher complex I activity but similar complex III activity relative to those of the offspring of dams fed with the basal diet.

### 3.4. Effects of Maternal Serine Deficiency on ROS Levels in Weanling Offspring Subjected to Diquat Challenge

**Figure 5:**
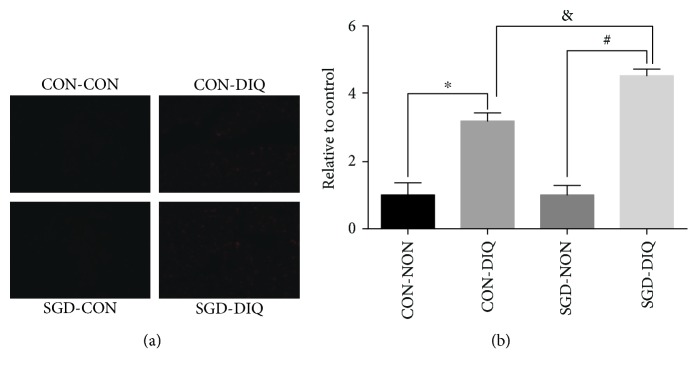
ROS content in the liver. ROS: reactive oxygen species; CON-NON: offspring from dams fed the basal diet; CON-DIQ: offspring from dams fed the basal diet and subjected to diquat challenge; SGD-CON: offspring from dams fed with serine-deficient diet; SGD-DIQ: offspring from dams fed with serine-deficient diet and subjected to diquat challenge. Values are expressed as LSmean plus pooled SEM, *n* = 8. ^∗^Mean values were significantly different between CON-NON and CON-DIQ (*P* < 0.05). ^#^Mean values were significantly different between SGD-NON and SGD-DIQ (*P* < 0.05). ^&^Mean values were significantly different between CON-DIQ and SGD-DIQ (*P* < 0.05).

As shown in Figure 5, maternal serine deficiency did not affect ROS levels in weanling offspring. However, under diquat challenge, ROS levels were significantly higher in the offspring of dams fed with the serine-deficient diet when compared with the offspring of dams fed with the basal diet.

### 3.5. Effects of Maternal Serine Deficiency on LPO and NADPH Levels and the Expression of *Hspd1* and *Hspa1a* in Weanling Offspring Subjected to Diquat Challenge

**Figure 6:**
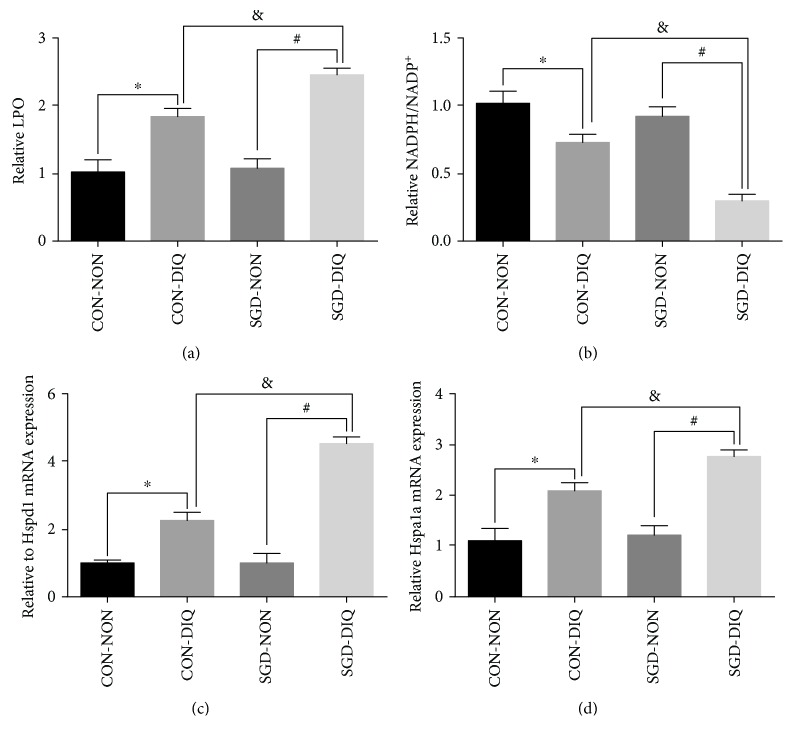
The level of LPO and NADPH and the expression of *Hspd1* and *Hspa1a* in the liver. LPO: lipid hydroperoxide; NADPH: nicotinamide adenine dinucleotide phosphate; *Hspd1*: heat shock protein 1; *Hspa1a*: heat shock protein 1A; CON-NON: offspring from dams fed the basal diet; CON-DIQ: offspring from dams fed the basal diet and subjected to diquat challenge; SGD-CON: offspring from dams fed with serine-deficient diet; SGD-DIQ: offspring from dams fed with serine-deficient diet and subjected to diquat challenge. Values are expressed as LSmean plus pooled SEM, *n* = 8. ^∗^Mean values were significantly different between CON-NON and CON-DIQ (*P* < 0.05). ^#^Mean values were significantly different between SGD-NON and SGD-DIQ (*P* < 0.05). ^&^Mean values were significantly different between CON-DIQ and SGD-DIQ (*P* < 0.05).

As shown in Figure 6, maternal serine deficiency did not affect LPO and NADPH levels and the expression of *Hspd1* and *Hspa1a* in weanling offspring. However, under diquat challenge, offspring of dams fed with the serine-deficient diet showed significantly higher LPO levels and expression of *Hspd1* and *Hspa1a* and significantly lower NADPH/NADP^+^ levels when compared with the offspring of dams fed with the basal diet.

### 3.6. Effects of Maternal Serine Deficiency on the Akt/AMPK/Sirt1 Pathway in Weanling Offspring Subjected to Diquat Challenge

**Figure 7:**
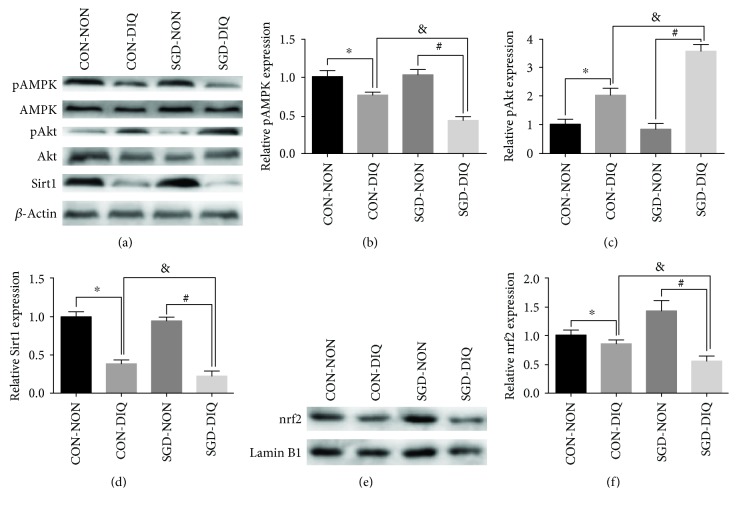
Expression of phosphorylated AMPK and Akt, Sirt1, and nuclear nrf2 in the liver. CON-NON: offspring from dams fed the basal diet; CON-DIQ: offspring from dams fed the basal diet and subjected to diquat challenge; SGD-CON: offspring from dams fed with serine-deficient diet; SGD-DIQ: offspring from dams fed with serine-deficient diet and subjected to diquat challenge. Values are expressed as LSmean plus pooled SEM, *n* = 8. ^∗^Mean values were significantly different between CON-NON and CON-DIQ (*P* < 0.05). ^#^Mean values were significantly different between SGD-NON and SGD-DIQ (*P* < 0.05). ^&^Mean values were significantly different between CON-DIQ and SGD-DIQ (*P* < 0.05).

As shown in Figure 7, maternal serine deficiency did not affect the expression of phosphorylated AMPK and Akt, Sirt1, and nuclear nrf2 in weanling offspring. However, under diquat challenge, offspring from dams fed with serine-deficient diet showed significantly lower expression of phosphorylated AMPK, Sirt1, and nrf2 but significantly higher phosphorylated Akt levels relative to those of the offspring of dams fed with the basal diet.

## 4. Discussion

Intrauterine malnutrition-induced stress during gestation is a major cause of diseases, such as diabetes, obesity, and insulin resistance in adult life [21]. Offspring of dams fed with suboptimal diet are often characterized by reduced antioxidant capacity (low GSH, CAT, and SOD concentrations) and increased levels of oxidant indices (lipid peroxidation and ROS) [22]. Oxidative stress programming could be regulated directly or indirectly by modulating gene expression through certain signaling pathways.

Serine is a nonessential amino acid that has been proven to be functionally essential because of its critical roles in the maintenance of cellular antioxidant status, cell survival, and cell proliferation. In the present study, our findings showed that the antioxidant abilities and oxidant indices of weanling offspring were not influenced by maternal serine deficiency, although serine-deficient diet caused significant decrease in serine and glycine content in both serum and liver in dams and their offspring. These results suggested that decreased serine and glycine content does not affect the activity and expression of antioxidant enzymes as well as GSH content in mice under normal condition. However, under diquat challenge, offspring of the dams fed with serine-deficient diet showed a significant downregulation of GSH, CAT, and SOD levels in both serum and the liver samples when compared with the offspring of dams fed with the basal diet. Further analysis confirmed the reduced antioxidant capacity in offspring of dams fed with the serine-deficient diet based on the observed downregulation of the expression of genes encoding the antioxidant enzymes. Additionally, GST activity exerts critical effects in cellular protection against oxidative damage and exogenous toxic chemicals including diquat. It detoxifies oxidized lipid and DNA by catalyzing the conjugation of GSH with electrophiles that are products of oxidative stress [23, 24]. As expected, under diquat challenge, GST activity in the liver samples was downregulated in offspring of the dams fed with serine-deficient diet when compared with those in the offspring of dams fed with the basal diet. The above results suggested that although maternal serine deficiency during pregnancy and lactation did not affect basal oxidative status in offspring, the offspring became vulnerable to oxidative challenges.

Diquat challenge triggered the reaction between superoxide anion radicals with oxygen molecules, leading to ROS production [25]. High ROS levels were observed in the livers of offspring subjected to diquat challenge. Moreover, ROS levels were considerably higher in the offspring of dams fed with the serine-deficient diet. These results were consistent with the dramatic downregulation of antioxidant enzymes, which further indicated that offspring of dams fed with the serine-deficient diet had more vulnerable antioxidant defense systems. Excessive ROS accumulation further causes oxidative damage to lipids, primarily in the form of lipid peroxidation [26]. The relatively higher MDA and LPO levels confirmed the greater lipid peroxidation in offspring of dams fed with the serine-deficient diet, consistent with the observed upregulation of ROS levels. Furthermore, relatively higher expression levels of *Hspd1* (a key mitochondrial stress marker) and *Hspa1a* (a cellular oxidative stress marker) were observed in the livers of offspring of dams fed with serine-deficient diet. The above results further confirmed that the livers of offspring of dams fed with serine-deficient diet were subjected to high oxidative stress. The glutathione and thioredoxin antioxidant systems consume NADPH as a cofactor for neutralizing ROS. Therefore, NADPH availability is a critical factor that affects the strength of the cellular response to oxidative stress [14]. Low NADPH levels were observed in the livers of offspring subjected to diquat challenge. Moreover, NADPH levels were considerably lower in the offspring of dams fed with the serine-deficient diet. Considering that serine is a major component required for cellular synthesis of NADPH [9, 10, 27], we speculated that maternal serine deficiency may influence NADPH availability in offspring when subjected to oxidative challenges. The lower serine and glycine content in both serum and liver in dams fed with the serine-deficient diet and their offspring provides further evidences for this speculation. As a result, inadequate NADPH availability could not neutralize the overaccumulated ROS caused by diquat. This may the possible mechanism that diquat reduced the activity and the expression of all antioxidant enzymes tested in the current study in the offspring of dams fed with the serine-deficient diet.

The mitochondrial complexes I and III are generally considered to be the primary sites of ROS production [28]. Particularly, growing evidence showed that complex I is very sensitive to oxidative stress and most of the free radical oxygen is generated by complex I [29, 30]. Only complex I, and not complex III, showed strong activity in offspring subjected to diquat challenge, thereby suggesting that the mitochondrial complex I is the primary site of ROS production under diquat challenge. Furthermore, complex I activity was higher in the offspring of dams fed with the serine-deficient diet when compared to that of the offspring of dams fed with the basal diet. The above results indicated that maternal serine deficiency can influence complex I activity and cause ROS overaccumulation in offspring subjected to oxidative damage.

Oxidative stress leads to inactivation of AMPK and Sirt1 pathways but results in the activation of the PI3K/Akt pathway. Oxidative challenge caused a significant increase in phosphorylated Akt levels but a significantly decrease in Sirt1 and phosphorylated AMPK levels in the offspring of dams fed with the serine-deficient diet, thereby suggesting that the impaired antioxidant defense system caused by maternal serine deficiency was mediated by a dysfunctional Akt/AMPK/Sirt1 pathway. Moreover, the downregulated expression of nuclear nrf2 further suggested that the Akt/AMPK/Sirt1 pathway plays a critical role in oxidative response by modulating its downstream target nrf2.

## 5. Conclusions

Our results showed that maternal serine deficiency did not affect basal oxidative status in weanling offspring. However, the weanling offspring were more vulnerable to oxidative challenges. Further, our results suggested that the dysfunctional antioxidant systems in offspring of dams fed with the serine-deficient diet were caused by lower NADPH availability and the dysfunctional Akt/AMPK/Sirt1 pathway potentially mediates the effects of maternal serine deficiency on offspring.

## Data Availability

The data used to support the findings of this study are available from the corresponding author upon request.

## Supplementary Materials

Supplementary Materials Supplementary Table 1: components of the control diet and serine- and glycine-deficient (SGD) diet used in the experiments. These two diets were purchased from Research Diets (New Brunswick, NJ, USA). Supplementary Table 2: sequences of primers used for RT-qPCR in the experiments. Gpx: glutathione peroxidase; SOD: superoxide dismutase; CAT: catalase; Hsp: heat shock protein.

## References

[1] Barker D. J., Winter P. D., Osmond C., Margetts B., Simmonds S. J. (1989). Weight in infancy and death from ischaemic heart disease. *Lancet*.

[2] Tarry-Adkins J. L., Fernandez-Twinn D. S., Chen J. H. (2016). Poor maternal nutrition and accelerated postnatal growth induces an accelerated aging phenotype and oxidative stress in skeletal muscle of male rats. *Disease Models & Mechanisms*.

[3] Ducker G. S., Rabinowitz J. D. (2017). One-carbon metabolism in health and disease. *Cell Metabolism*.

[4] Mattaini K. R., Sullivan M. R., Vander Heiden M. G. (2016). The importance of serine metabolism in cancer. *The Journal of Cell Biology*.

[5] Zhou X., He L., Zuo S. (2018). Serine prevented high-fat diet-induced oxidative stress by activating AMPK and epigenetically modulating the expression of glutathione synthesis-related genes. *Biochimica et Biophysica Acta (BBA) - Molecular Basis of Disease*.

[6] Kim D. H., Park C. H., Park D. (2014). Ginsenoside Rc modulates Akt/FoxO1 pathways and suppresses oxidative stress. *Archives of Pharmacal Research*.

[7] Murata H., Ihara Y., Nakamura H., Yodoi J., Sumikawa K., Kondo T. (2003). Glutaredoxin exerts an antiapoptotic effect by regulating the redox state of Akt. *The Journal of Biological Chemistry*.

[8] Caito S., Rajendrasozhan S., Cook S. (2010). SIRT1 is a redox-sensitive deacetylase that is post-translationally modified by oxidants and carbonyl stress. *The FASEB Journal*.

[9] Ducker G. S., Chen L., Morscher R. J. (2016). Reversal of cytosolic one-carbon flux compensates for loss of the mitochondrial folate pathway. *Cell Metabolism*.

[10] Lewis C. A., Parker S. J., Fiske B. P. (2014). Tracing compartmentalized NADPH metabolism in the cytosol and mitochondria of mammalian cells. *Molecular Cell*.

[11] Tedeschi P. M., Markert E. K., Gounder M. (2013). Contribution of serine, folate and glycine metabolism to the ATP, NADPH and purine requirements of cancer cells. *Cell Death & Disease*.

[12] Martinez-Reyes I., Chandel N. S. (2014). Mitochondrial one-carbon metabolism maintains redox balance during hypoxia. *Cancer Discovery*.

[13] Yang M., Vousden K. H. (2016). Serine and one-carbon metabolism in cancer. *Nature Reviews Cancer*.

[14] Zhang T., Gillies M. C., Madigan M. C. (2018). Disruption of de novo serine synthesis in Müller cells induced mitochondrial dysfunction and aggravated oxidative damage. *Molecular Neurobiology*.

[15] Zhou X., He L., Wu C., Zhang Y., Wu X., Yin Y. (2017). Serine alleviates oxidative stress via supporting glutathione synthesis and methionine cycle in mice. *Molecular Nutrition & Food Research*.

[16] Zhou X. H., Zhang Y. M., He L. Q. (2017). Serine prevents LPS-induced intestinal inflammation and barrier damage via p53-dependent glutathione synthesis and AMPK activation. *Journal of Functional Foods*.

[17] Cao G., Tao F., Xin L., Li Z., Zhou X. (2018). Effects of maternal serine supplementation on high-fat diet-induced oxidative stress and epigenetic changes in promoters of glutathione synthesis-related genes in offspring. *Journal of Functional Foods*.

[18] Maddocks O. D., Berkers C. R., Mason S. M. (2013). Serine starvation induces stress and p53-dependent metabolic remodelling in cancer cells. *Nature*.

[19] Chen R., Zou Y., Mao D. (2014). The general amino acid control pathway regulates mTOR and autophagy during serum/glutamine starvation. *The Journal of Cell Biology*.

[20] Jin X. L., Wang K., Li Q. Q. (2017). Antioxidant and anti-inflammatory effects of Chinese propolis during palmitic acid-induced lipotoxicity in cultured hepatocytes. *Journal of Functional Foods*.

[21] Saad M. I., Abdelkhalek T. M., Haiba M. M. (2016). Maternal obesity and malnourishment exacerbate perinatal oxidative stress resulting in diabetogenic programming in F1 offspring. *Journal of Endocrinological Investigation*.

[22] Hracsko Z., Orvos H., Novak Z., Pal A., Varga I. S. (2008). Evaluation of oxidative stress markers in neonates with intra-uterine growth retardation. *Redox Report*.

[23] Hayes J. D., Strange R. C. (2000). Glutathione S-transferase polymorphisms and their biological consequences. *Pharmacology*.

[24] Hayes J. D., Strange R. C. (1995). Potential contribution of the glutathione S-transferase supergene family to resistance to oxidative stress. *Free Radical Research*.

[25] Farrington J. A., Ebert M., Land E. J., Fletcher K. (1973). Bipyridylium quaternary salts and related compounds. V. Pulse radiolysis studies of the reaction of paraquat radical with oxygen. Implications for the mode of action of bipyridyl herbicides. *Biochimica et Biophysica Acta (BBA) - Bioenergetics*.

[26] James S. J., Cutler P., Melnyk S. (2004). Metabolic biomarkers of increased oxidative stress and impaired methylation capacity in children with autism. *The American Journal of Clinical Nutrition*.

[27] Gao X., Lee K., Reid M. A. (2018). Serine availability influences mitochondrial dynamics and function through lipid metabolism. *Cell Reports*.

[28] Goguadze N., Zhuravliova E., Morin D., Mikeladze D., Maurice T. (2017). Sigma-1 receptor agonists induce oxidative stress in mitochondria and enhance complex I activity in physiological condition but protect against pathological oxidative stress. *Neurotoxicity Research*.

[29] Ott M., Gogvadze V., Orrenius S., Zhivotovsky B. (2007). Mitochondria, oxidative stress and cell death. *Apoptosis*.

[30] Lenaz G., Bovina C., D'Aurelio M. (2002). Role of mitochondria in oxidative stress and aging. *Annals of the New York Academy of Sciences*.

