# Supplementary material for: Weanling Offspring of Dams Maintained on Serine-Deficient Diet Are Vulnerable to Oxidative Stress
Source: Oxid Med Cell Longev. 2018 Sep 16;2018:8026496. doi: 10.1155/2018/8026496 (PMC6165615; doi:10.1155/2018/8026496)
Supplement: Supplementary Materials — Supplementary Table 1: components of the control diet and serine- and glycine-deficient (SGD) diet used in the experiments. These two diets were purchased from Research Diets (New Brunswick, NJ, USA). Supplementary Table 2: sequences of primers used for RT-qPCR in the experiments. Gpx: glutathione peroxidase; SOD: superoxide dismutase; CAT: catalase; Hsp: heat shock protein. [file 8026496.f1.docx]

**Supplementary table 1** Components of the control diet, and serine and glycine deficient (SGD) diet used in the experiments. These two diets were purchased from Research Diets (New Brunswick, NJ, USA).

**Supplementary Table 2** Sequences of primers used for RT-qPCR in the experiments. Gpx, glutathione peroxidase; Sod, superoxide dismutase; Cat, catalase; Hsp, heat shock protein.

**Supplementary table 1** Diet components.

| Components | Control diet, gm | SGD diet, gm |
| --- | --- | --- |
| L-Arginine | 10 | 10 |
| L-Histidine-HCl-H2O | 6 | 6 |
| L-Isoleucine | 8 | 8 |
| L-Leucine | 12 | 12 |
| L-Lysine-HCl | 14 | 14 |
| L-Methionine | 6 | 6 |
| L-Phenylalanine | 8 | 8 |
| L-Threonine | 8 | 8 |
| L-Tryptophan | 2 | 2 |
| L-Valine | 8 | 8 |
| L-Alanine | 10 | 10 |
| L-Asparagine-H2O | 5 | 5 |
| L-Aspartate | 10 | 10 |
| L-Cystine | 4 | 4 |
| L-Glutamic Acid | 30 | 30 |
| L-Glutamine | 5 | 5 |
| L-Glycine | 10 | 0 |
| L-Proline | 5 | 5 |
| L-Serine | 5 | 0 |
| L-Tyrosine | 4 | 4 |
| Corn Starch | 550.5 | 565.5 |
| Maltodextrin 10 | 125 | 125 |
| Cellulose | 50 | 50 |
| Corn Oil | 50 | 50 |
| Mineral Mix S10001 | 35 | 35 |
| Sodium Bicarbonate | 7.5 | 7.5 |
| Vitamin Mix V10001 | 10 | 10 |
| Choline Bitrartrate | 2 | 2 |
| Total | 1000 | 1000 |

**Supplementary Table 2** Sequences of primers used for RT-qPCR.

| Gene | 5’-3’ Primer sequence |
| --- | --- |
| Gpx1 | F: AGTCCACCGTGTATGCCTTCT |
|  | R: GAGACGCGACATTCTCAATGA |
| Gpx4 | F: GCAACCAGTTTGGGAGGCAGGAG |
|  | R: CCTCCATGGGACCATAGCGCTTC |
| Sod1 | F: AACCAGTTGTGTTGTCAGGAC |
|  | R: CCACCATGTTTCTTAGAGTGAGG |
| Sod2 | F: AACCAGTTGTGTTGTCAGGAC |
|  | R: CTCGGTGGCGTTGAGATTGTT |
| Cat | F: CCTCGTTCAGGATGTGGTTT |
|  | R: TCTGGTGATATCGTGGGTGA |
| Hspd1 | F: TGATGTTGGCTGTGGATGCT |
|  | R: GACACCCTTTCTTCCAACCTTT |
| Hspa1a | F: CAAGAACGCGCTCGAATCCTA |
|  | R: TCCTGGCACTTGTCCAGCAC |
| β-actin | F: TGTCCACCTTCCAGCAGATGT |
|  | R: AGCTCAGTAACAGTCCGCCTAGA |
